# Transcriptome analysis of alternative splicing in the pathogen life cycle in human foreskin fibroblasts infected with *Trypanosoma cruzi*

**DOI:** 10.1038/s41598-020-74540-9

**Published:** 2020-10-15

**Authors:** Hyeim Jung, Seonggyun Han, Younghee Lee

**Affiliations:** 1 Department of Internal Medicine, Washington University School of Medicine, St. Louis, MO 63110 USA; 2 Department of Biomedical Informatics, University of Utah School of Medicine, Salt Lake City, UT 84108 USA

**Keywords:** Gene expression, Data integration, Genome informatics, Parasite genomics

## Abstract

*Trypanosoma cruzi* is an intracellular protozoan parasite that causes Chagas disease as a zoonotic pathogen. The parasite has been shown to remodel expression in the host transcriptome under different conditions. Although alternative splicing (AS) is involved in virtually every biological function in eukaryotes, including cellular differentiation and responses to immune reactions, host AS events that occur as a result of *T. cruzi* infection have yet to be explored. In this study, we bioinformatically investigated the transcriptome AS dynamics of *T. cruzi* (Y strain) infected human foreskin fibroblasts using RNA-Seq data captured over four timepoints (4, 24, 48, and 72 h post infection (hpi)). We identified 1768, 399, 250, and 299 differentially expressed exons (AS exons) at 4, 24, 48, and 72 hpi, respectively, showing that host AS mechanism may have a significant role in the intracellular life cycle of the parasite. We present an exon skipping event in *HDAC7*, which is a candidate gene that is important in the parasite’s cell cycle. To sum up, this bioinformatics analysis of transcriptome may provide new potential insight into AS regulation in human foreskin fibroblast (HFF) cells infected by *T. cruzi* and into its implication to the parasite life cycle. Moreover, identified AS genes may provide new potential molecular candidates for improving treatment.

## Introduction

The protozoan parasite *Trypanosoma cruzi* causes Chagas disease (kissing bug disease). It is a zoonotic pathogen that can be transmitted by triatomine insects to a wide range of mammalian hosts including human and domestic animals^1^. Chagas disease can lead to acute inflammation in many tissues including the skin, heart, and intestinal tract^2^. Interestingly, 94.5% of symptomatic human patients have a heart problem in the chronic stage^3^. Although this disease has been discovered over 100 years ago and endemic in a large portion of Latin America (i.e. 6–7 million people infected), it is now becoming a global health problem due to a large migration from Latin America to other countries, including North America^4^.

*Trypanosoma cruzi* is an intracellular parasite with a complex life cycle that replicates and survives in diverse nucleated host cells including those of the heart and intestinal tract. Briefly, when the triatomine bug bites a vertebrate’s skin for blood-feeding, the parasite passes into the host’s bloodstream as metacyclic trypomastigotes. These invade various nucleated cells as amastigotes, proliferate in cytosol of the cells for 3–5 days, and then differentiate into trypomastigotes, lysing the host cells in order to release themselves^5^. Intracellular parasites interact with their host cells to replicate themselves and survive in host cells, although it remains unclear how the modifications in the host cell contribute to the parasite’s growth. Thus, to advance infection interventions, it is essential to understand what functional processes of the host cells are affected by the parasite to enable their transmission and host colonization^6,7^. Accordingly, many studies have characterized mechanisms of interaction between parasite and host cell to identify essential processes. Processes that have been focused upon for development of effective treatment are adhesion to the host cell, molecular mechanisms for escaping from lysosomes, and cell cycle in the host cell^7–12^. A recent study showed that the parasite also remodels the host cell transcriptome under different conditions^13–18^. In particular, genes related to energy metabolism, immune response, and cell cycle were significantly remodeled at the transcript level in infected human foreskin fibroblasts (HFFs), and the remodeled genes were also enriched in functional pathways (i.e. cell cycle process) that are important to the parasite’s life cycle in HFFs^19,20^. Therefore, investigating dynamic transcriptomic profiles of infected host cells can confer new insights into molecular mechanisms underlying the parasite’s life cycle and potentially identify new molecular targets for improved treatments.

Alternative splicing (AS) is a key method for regulating genes, accounting for the biological complexity of various cell types. In eukaryotes, more than 90% of genes undergo AS. Selective usage of exons can generate multiple proteins from a single gene^21^, and splicing mechanisms can produce tissue-specific isoforms^22^. Furthermore, many genes that function in the cell cycle and immune response also have alternative isoforms. Post-transcriptional processes and mRNA splicing are known to occur with developmental transitions in trypanosomes^23–27^. In addition, alteration of alternative splicing in host human cells was observed during parasite infection, suggesting it plays a role in the infection^28^. That is, the functional pathways important for the *T. cruzi* life cycle are potentially regulated by AS. However, genome-wide analysis of AS in host cells infected with *T. cruzi* has not yet to be performed.

In this study, we analyzed previously published RNA-Seq data^20,29^ from HFF cells infected with *T. cruzi* (Y strain) and identified AS events across a time series (i.e. 4 h post infection (hpi), 24 hpi, 48 hpi, and 72 hpi) through bioinformatics analysis.

## Results

### Dynamic changes of alternative splicing occur in human foreskin fibroblasts infected with *T. cruzi*

**Figure 1:**
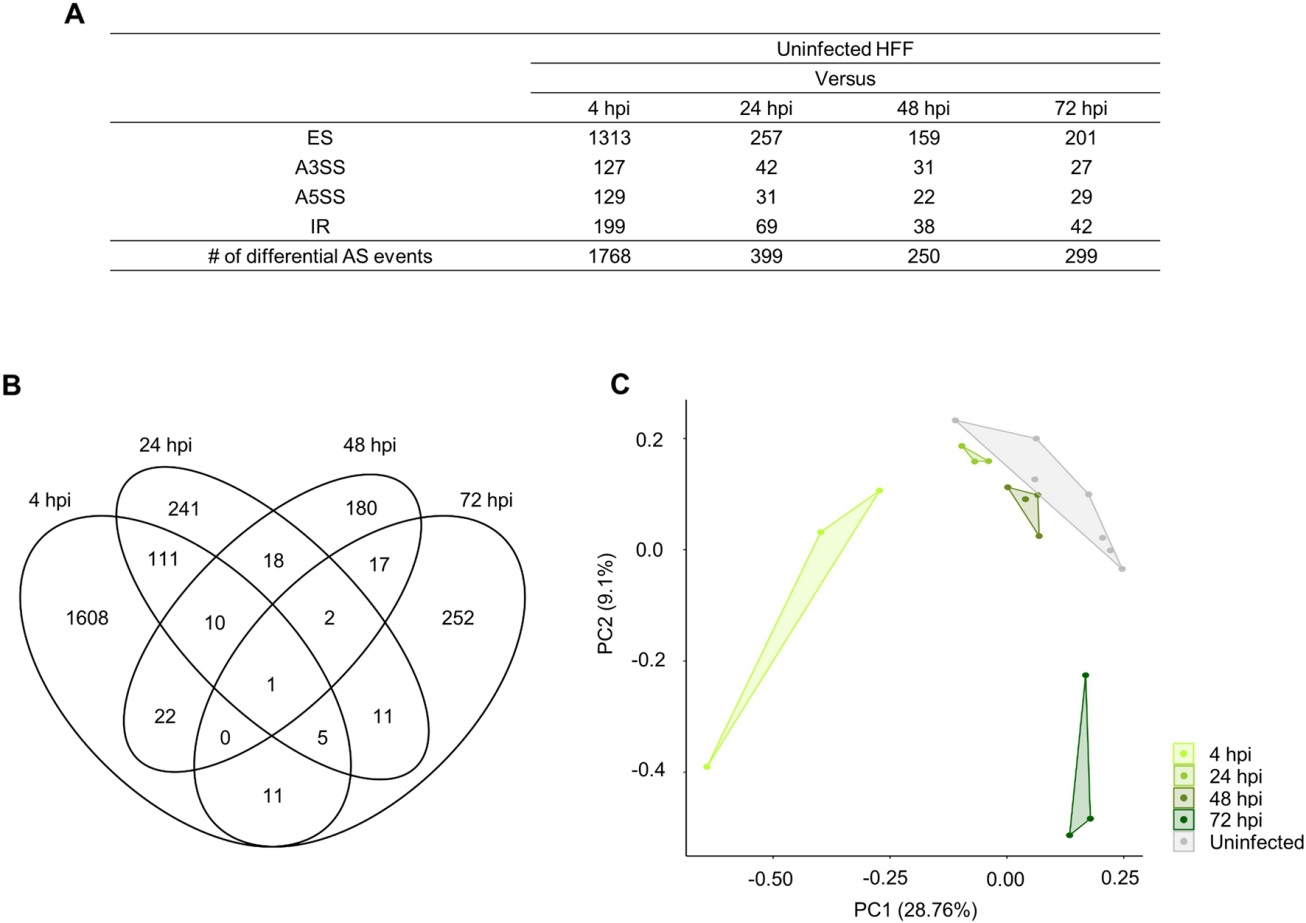
Differential AS events between infected and uninfected HFF cells. (**A**) Summary of identified AS exons by alternative splicing type; *ES* exon skipping, *A3SS* alternative 3′ splice site, *A5SS* alternative 5′ splice site, and *IR* intron retention in each infection time (4, 24, 48, or 72 hpi). (**B**) Venn diagram indicating overlaps of identified AS exons across infection phases. (**C**) Principal component analysis (PCA) of AS exons. Each dot indicates an individual sample. Grey indicates control uninfected samples; and green indicates infected samples.

To investigate global transcriptional response of host cells after *T. cruzi* infection, we identified exons that were differentially spliced between uninfected and *T. cruzi* infected HFFs. We considered four major types of splicing events: exon skipping, alternative 5′ and 3′ splicing, and intron retention. We identified 1325, 348, 224, and 249 genes with significantly differentially expressed AS exons (i.e. 1768, 399, 250, and 299 AS events) at 4, 24, 48, and 72 hpi, respectively (Fig. 1A,B). The AS exons identified for each infection phase are summarized in Supplementary Table S1.

We observed the greatest number of AS events (1768) at early infection (4 hpi), and 1608 (91.0%) of those events were unique to the 4 hpi stage. Of the other 9% (160 events) of AS events, 127 were also present at 24 hpi, and accounted for 31.8% of the 399 AS events identified at 24 hpi. However, only 10 AS events remained differentially expressed at 4, 24, and 48, and one AS event at all 4, 24, 48, and 72 hpi (Fig. 1B). That is, these changes were dynamic between infection phases; only a very small number of AS events were detected in multiple infection phases, suggesting that the majority are phase specific.

### Phase stratification by AS exons

We performed PCA analysis on exon percent spliced in (PSI) values to verify whether the four different infection phases can be differentiated by AS profiles. When all AS events were considered, infected samples did not separate from uninfected control samples (Supplementary Fig. S1). However, when considering only significantly different AS events, PCA analysis not only successfully separated infected samples from uninfected but also stratified the infected samples by infection phase (Fig. 1C). These results support that infection with *T. cruzi* might remodel AS events in HFF transcripts according to the infection stages.

### Functional enrichment analysis of AS genes at each infection phase

**Figure 2:**
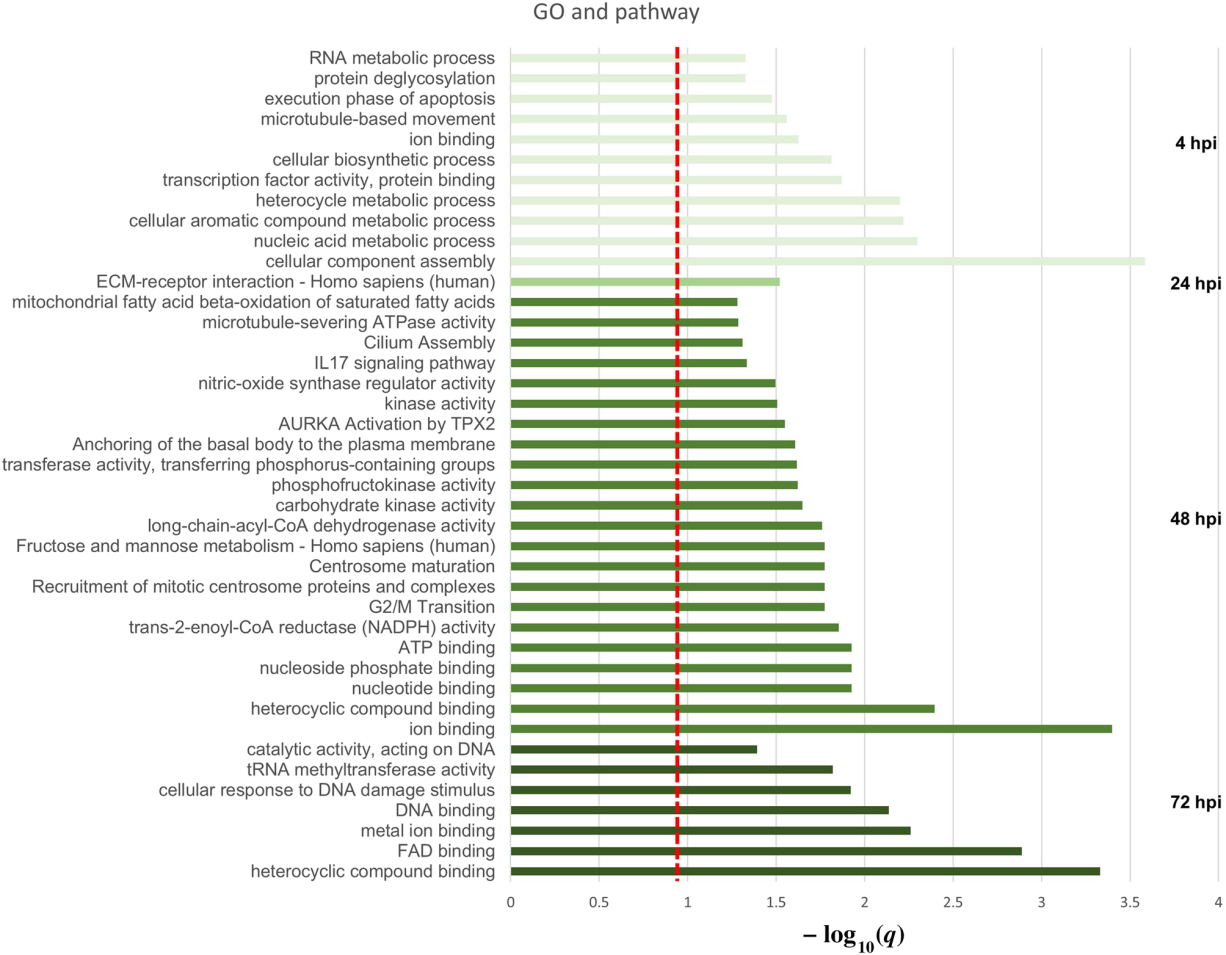
GO terms and pathways overrepresented in AS genes at each infection phase. The red line represents the FDR significance cutoff of *q* = 0.05 and the x-axis is − log_10_(*q*).

We used gene enrichment analysis to explore the molecular functions of genes with differential AS at each phase of *T. cruzi* infection. The significant gene ontology (GO) terms and pathway results are summarized in Fig. 2 and Supplementary Table S2.

### 4 hpi

The GO and pathways most significantly enriched in the early stage (*q* < 0.05) are related to key initial infection processes such as cell binding and lysosome escape. Consistent with prior study of differentially expressed genes^20^, we found early phase AS genes to be enriched in GO terms related to protein synthesis, including “cellular component assembly”, “transcription factor activity”, and “RNA metabolic process”. Other significant enrichments include terms related to a lipid remodeling of proteins and membranes that occurs during early infection, including “ion binding”, “cellular biosynthetic process”, “execution phase of apoptosis”, “cellular nitrogen compound metabolic process”, and “nucleic acid metabolic process”. These may reflect dynamic perturbations of biological pathways involved in metabolism and lipid biosynthesis to support an increasing intracellular parasite burden. Interestingly, our study also identified “microtubule-based movement” as significantly enriched, which process is important for early infection^30,31^. This GO term was not identified when considering only differentially expressed genes^20^. At early phase, host lysosomes move to the site of parasite entry and construct a parasitophorous vacuole (PV), within which the parasite is protected and amplifies itself. Microtubules and kinesin are required for the migration of lysosomes from the perinuclear region to the cell periphery^32–34^. Therefore, this remodeling of AS events in early infected cells might be implicated in PV formation.

### 24 hpi

We identified no significantly enriched GO terms at 24 hpi that met our criteria (*q* < 0.05); however, the term “oxidoreductase activity, acting on the CH–CH group of donors” was marginally significant (*p* = 0.000739 and *q* = 0.06498). After the early stage, lipid biosynthesis is highly activated for parasite development and puts the host cell under oxidative stress, which it may respond to by modulating these genes^19,35,36^. Furthermore, we identified significant enrichment in “ECM-receptor interaction”, as consistent with that *T. cruzi* regulates the extracellular matrix (ECM) to invade cells, enhancing infection at the early-mid phase^37^. That is, remodeling of AS patterns at 24 hpi may facilitate the interaction between host and parasite.

### 48 hpi

At 48 hpi, AS genes were significantly enriched in many GO terms and pathways related to energy metabolism and cell cycle. Terms relating to energy metabolism included: “nucleoside phosphate binding”, “ATP binding”, “transferase activity, transferring phosphorus-containing groups”, “kinase activity”, “fructose and mannose metabolism”, and “phosphofructokinase activity”. Terms related to the cell cycle included “G2/M transition”, “centrosome maturation”, “recruitment of mitotic centrosome proteins and complexes”, and “regulation of PLK1 activity at G2/M transition”. Other enriched terms included “IL17 signaling pathway” and “cilium assembly”. Energy metabolism and the cell cycle are important to later infection stages to fuel the parasite because *T. cruzi* represses host cell proliferation in order to allow its own amplification. In the previous DEG study, researchers expected that the burden of replicating parasites may cause dramatic changes in expression of host energy homeostasis genes; however, they did not find significant enrichment of energy metabolism among differentially expressed genes. Thus, they suggested that genes related to energy metabolism may be controlled at a post-transcriptional level^19^. Our findings suggest that genes related to energy metabolism may be regulated by AS in *T. cruzi* infected cells.

### 72 hpi

At 72 hpi, there were no significantly enriched pathways (*q* < 0.05); however, some cell cycle pathways were marginally significant, such as “regulation of PLK1 Activity at G2/M Transition” (*p* < 0.005046, *q* < 0.180) and “G2/M Transition” (*p* < 0.0071, *q* < 0.180).

Taken together, genes with differential AS in *T. cruzi* infected HFF cells may be implicated in nucleic acid metabolism, lipid biosynthesis or cell–cell interactions at early-mid phase (from 4 to 24 hpi); at later stages (from 48 to 72 hpi), AS genes may have significant roles in cell cycle and energy metabolism. The significantly enriched GO terms and pathways for each phase are summarized in Supplementary Table S2.

### Longitudinal patterns in PSI of differential AS exons

Supplementary Figure S2 shows the hierarchical clustering of genes with AS exons by PSI values over time. Samples grouped well according to timepoints (4, 24, 48, and 72 hpi). In addition, we obtained seven sub-clusters through k-means grouping. Seven sub-clusters were identified (Supplementary Fig. S3), and the gene lists for each sub-cluster are summarized in Supplementary Table S3. We then performed functional analysis for the gene lists for each sub-cluster (Supplementary Table S4). For example, in the cluster 1, the upregulation was observed at 4 hpi and continued, then decreased at 72 hpi. These genes were overrepresented in DNA processing, including “DNA-binding transcription factor activity”, “RNA metabolic process”, and “gene expression”. It suggests such longitudinal patterns in PSI may be implicated in the increased replicating parasite burden. Also, “oxidoreductase activity, acting on the CH–CH group of donors” were enriched in the gene lists. As mentioned above, GO terms enriched at 24 hpi, the genes with this longitudinal pattern in PSI may also be involved in host cell under oxidative stress from highly activated lipid biosynthesis for parasite development. In the cluster 2, PSIs tend to turn to peak at 72 hpi. The genes in the cluster 2 were enriched in apoptosis-related pathways, such as “apoptotic nuclear changes “, “execution phase of apoptosis “, and “NACHT domain binding”. PSIs in the cluster 4 were relatively constant across the timelines. The genes in the cluster 4 were enriched in GO term of “GTPase activator activity”, which is related to a function required for intracellular infection overall. In the cluster 7, PSI levels tend to peak at 48 hpi, and those genes were enriched in GO term of “transferase activity, transferring phosphorus-containing groups”, which is related to energy metabolism as consistent to GO terms enriched at 48 hpi.

### Splicing factors associated with differential AS exons as candidate regulators

Testing for associations between splicing factor gene expression and AS PSI levels with cutoff criteria of FDR value < 0.05 and absolute correlation > 0.8 (see “Methods” section), we found 201 unique splicing factor genes (713 unique AS exons) that are candidate regulators for controlling our significant AS events. Thus, of all 280 known splicing factors, approximately 70% are statistically implicated in these differential splicing events. The top five most influential splicing factors were *SFPQ*, *PPIH*, *SF3B2*, *FUS*, and *SNRNP25* associating with 149, 139, 138, 124, and 119 AS exons, respectively (Supplementary Table S5).

### Case study: functional implications of HDAC7 exon 15 skipping

**Figure 3:**
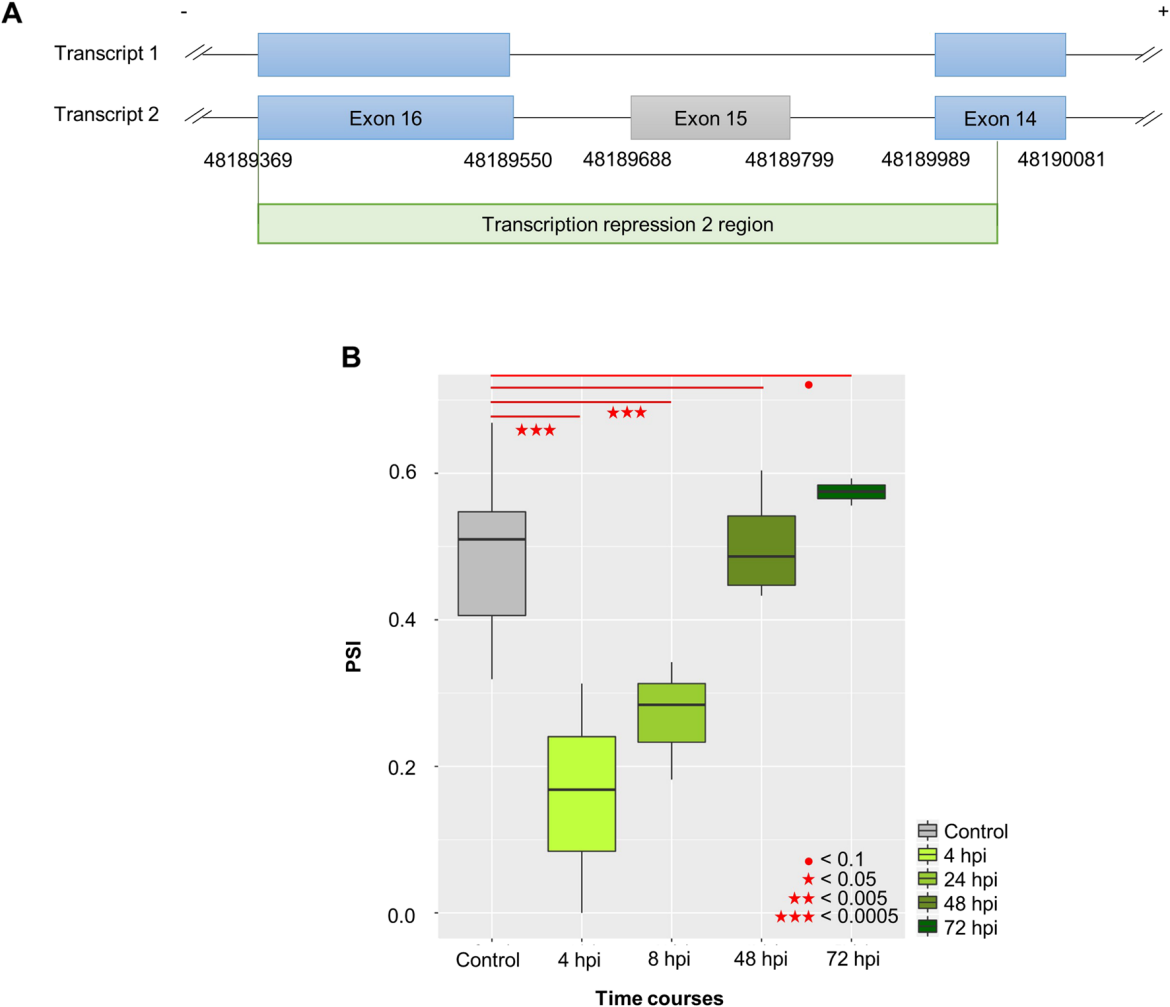
The functional impact of *HDAC7* exon 15 skipping. (**A**) Schematic of exon skipped (transcript 1) and canonical (transcript 2) transcript isoforms. Exon 15 skipping may lead to disruption of the transcription repression 2 region. (**B**) Boxplot showing exon inclusion level (PSI) by infection phase.

**Figure 4:**
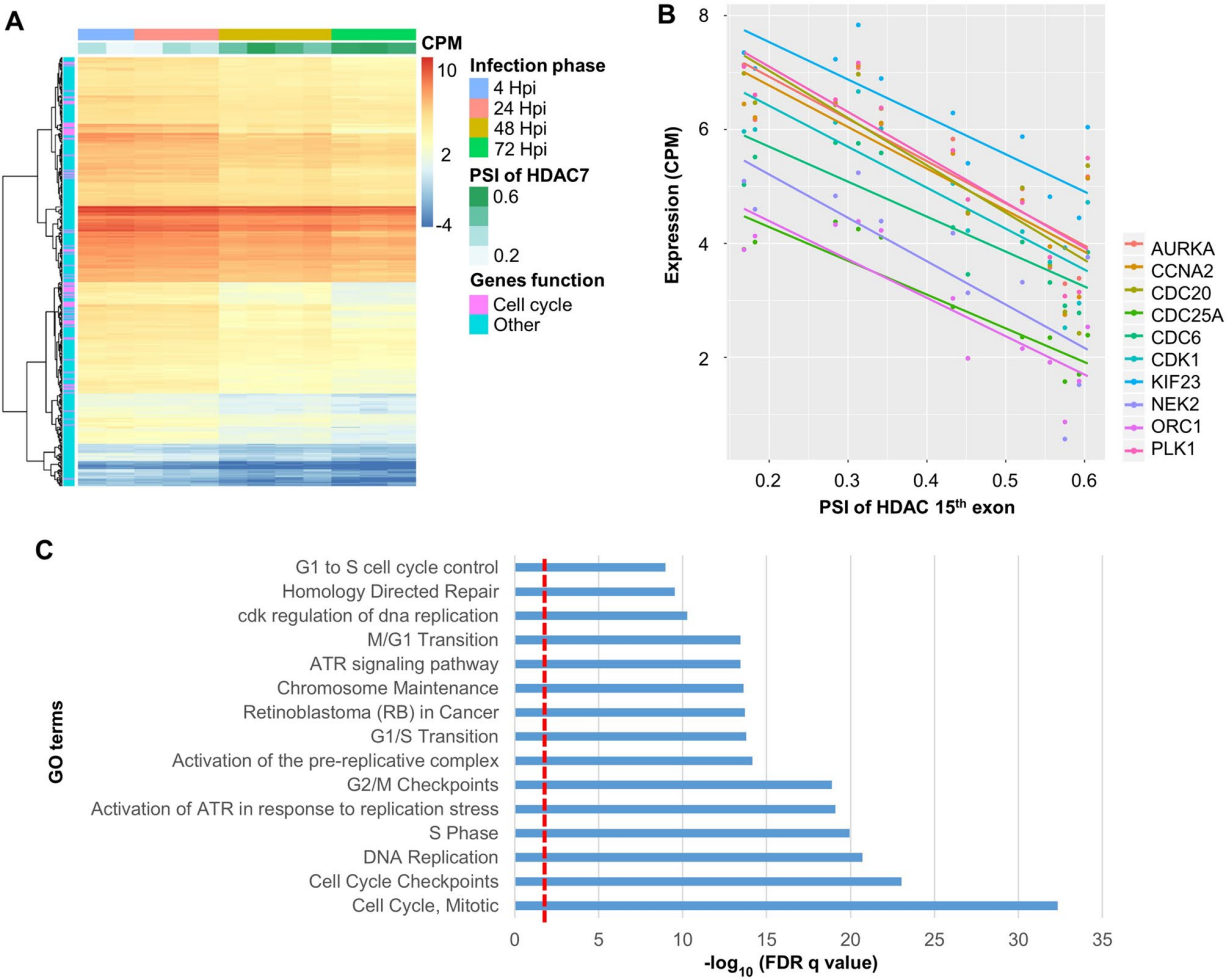
Gene expression profile and significant GO terms correlated with *HDAC7* exon 15 skipping. (**A**) Gene expression heatmap of 356 genes associated with exon 15 skipping of *HDAC7*. Higher expression is indicated in red, lower in blue. Each column is a sample and each row a gene. Columns are grouped by infection phase, and the first column is the percent spliced in (PSI) level of exon 15 of *HDAC7*. Purple annotation indicates cell cycle-related genes, blue genes with other functions. (**B**) Cell cycle genes negatively correlated with *HDAC7* PSI values. The x-axis is the *HDAC7* PSI level while the y-axis is the expression of the indicated gene. (**C**) GO terms significantly overrepresented in genes negatively correlated with exon 15 skipping (*q* < 0.05).

A recent study has revealed that *T. cruzi* infection modulates the expression of genes related to immune response and cell cycle function^19^. Especially, they found that the dynamic pattern of cell cycle genes such as *CDK1*, *AURKA*, and *PLK1* are upregulated in early infection (4 hpi), and this upregulation tends to peak at 24 hpi and then precipitously decrease. At 48 hpi, expression of these genes returns to a level similar to controls, then continues to decrease. At 72 hpi, these cell cycle genes were significantly downregulated^19^. This dynamic expression pattern suggests that these host cell cycle genes may be important to complete the pathogen intracellular life cycle during infection^19^. We identified AS genes with dynamic changes of exon expression that followed the same pattern observed in the previous study: an AS event significant at both 4 and 24 hpi, no difference at 48 hpi, and the opposite direction at 72 hpi. Only one AS exon fit these criteria, exon 15 of *HDAC7*. As shown in Fig. 3A, this skipping event is present in the reference annotation (i.e. GTF file based on GRCh37) and encodes the functional region “Transcription repression 2”. In other words, skipping level may denote the amount of dysfunctional *HDAC7* protein product, while the opposite indicates contrast. As shown in Fig. 3B, this exon was the most frequently skipped at both 4 hpi (*p* = 1.29e−07) and 24 hpi (*p* = 1.46e−05), but at 48 hpi (*p* = 0.809) the skipped level tended to be lower, returning to a level similar to the control group. And then, the skipping events continued to decrease until 72 hpi (*p* = 8.49e−02, marginal significant). That is, *HDAC7* is the only one gene with AS exon that has reverse correlation with cell cycle genes in gene expression. In other words, skipping level denotes the amount of dysfunctional *HDAC7* protein product, while the opposite indicates contrast. Most notably, *HDAC7* tends to be involved in regulating transcription factor binding to DNA by altering chromosome structure, suggesting that it is a candidate regulator of cell cycle genes. That is, there is possibility that dynamic change of cell cycle gene expression might be regulated by skipping of 15th exon of *HDAC7* (loss of the functional exon region). A previous study demonstrated that siRNA-mediated knockdown of *HDAC7* in vascular smooth muscle cells (SMCs) lead to increased cell proliferation^38,39^. Although our data was collected from HFFs rather than SMCs, skipping of *HDAC7* exon 15 may similarly result in abnormal function, and consequent increase in cell cycle functions. Therefore, with the assumption that higher skipping level of the 15th exon of *HDAC7* may account for more expression of genes involved in cell cycle, we performed a linear regression and calculated correlations between the inclusion level (PSI) of *HDAC7* 15th exon and each expression of the entire protein coding genes (20,758) in order to verify whether genes whose expression levels are associated with the exon skipping level have a functional role in cell cycles or not. As described in the Methods, gene expression values (CPM; count per million values) were determined by the original study^19^. We identified 522 genes significantly associated with *HDAC7* exon 15 skipping (FDR < 0.05 and absolute value of correlation coefficient > 0.9). Of these, 196 had positive correlations and 356 were negatively correlated. Surprisingly, the negatively correlated genes (i.e. positively correlated with skipping rate) were significantly overrepresented in pathways related to cell cycle function (Fig. 4), while the positively correlated genes were not. This suggests the aberrant transcript isoform of *HDAC7* with exon 15 skipping may be associated with a failure to repress cell cycle genes in infected HFF cells. Figure 4A summarizes the expression levels of negatively correlated genes as a heatmap. As mentioned above, the original study highlighted ten cell cycle genes (i.e. *CDK1*, *AURKA*, *PLK1,* and etc.) which were dynamically changed during infection phases^19^. Our study showed that all of the ten genes related to cell cycle were also associated with exon 15 PSI level (Fig. 4B).

## Discussion

This study analyzed systemic RNA-Seq data to reveal differential AS patterns between infected with *T. cruzi* and control HFF cells across infection phases (4, 24, 48, and 72 hpi). It is first designed to investigate genome-wide host AS events throughout intracellular infection of *T. cruzi*. Our functional enrichment study with identified AS genes at each timepoint suggested that remodeled AS have an important role in *T. cruzi* intracellular infection cycle. Our PCA and hierarchal cluster analysis separated three distinct groups: (1) 4 hpi, (2) 24 and 48 hpi, and (3) 72 hpi. The intracellular stages of *T. cruzi* are generally classified according to their maturation status into nascent (4 hpi) and mature replicative stages (24, 48, and 72 hpi), and the original publication that generated the RNA-seq data we analyzed in this study followed the same classification scheme and separated the timepoints into distinct phases-early (4 hpi), mid (24 and 48 hpi), and late (72 hpi)-based on their analysis, suggesting distinct characteristics in the host cell response by time post-infection. That is, using our phase stratification based on AS exons, we suggest that AS regulation may be an important mechanism contributing to unique characteristics of the host cell in each phase. In addition, *T. cruzi* has one of the most complex life cycles during intracellular infection, which means that alterations in AS exon expression (i.e. high or low frequency of splicing) may not be constant across the infection timeline.

The interactions of AS and gene expression can regulate each other. For example, some transcripts resulting from AS can undergo nonsense-mediated decay (NMD), and so expression of their genes may be decreased. Therefore, we further evaluated if the genes altered by AS had changes at the gene expression level at each hpi as described in the original studies. Among the genes identified to be differentially expressed (DEG) in the original studies^20,29^, a small number overlapped with our identified AS genes: 15 (1.2%), 25 (7.8%), 2 (1.0%), and 14 (5.6%) at 4, 24, 48, and 72 hpi, respectively. The small proportion of overlap at each timepoint suggests that AS and DEG might be differently regulated from each other.

Identifying and understanding the biological processes of genes affected by AS events that are changed at each phase could more effectively characterize the distinct features of each phase compared to the baseline. Therefore, we performed functional analysis on the genes that have identified differential AS exons at each timepoint. At early-mid infection (4 and 24 hpi), AS genes were overrepresented in the pathways related to functions for initial infection, such as lipid biosynthesis or cell–cell interactions. Moreover, the genes that had AS events present at both 4 and 24 hpi were moderately enriched in the pathway related to beta1 integrin signaling (4 hpi; *p* < 0.0083, 24 hpi; *p* < 0.0011). The beta 1 integrin family has been reported to be involved in the binding and entry of *T. cruzi* to human macrophages^40^. In addition, another study suggested that integrin family members may trigger host cell response to trypomastigotes^41^. Therefore, those early stage AS events may be a key factor for the interaction between *T. cruzi* and the host cell. These AS genes could be potential candidates for the development of inhibitors that block trypomastigote binding. In contrast to early phase, the AS genes identified at 48 and 72 hpi were enriched in cell cycle and energy metabolism, and these AS genes may be used for completing the parasite replication. For 4, 48, and 72 hpi, the ion metabolism pathway was overrepresented (4 hpi; *q* < 0.023, 24 hpi; *q* < 0.1602 (*p* < 0.0050), 48 hpi; *q* < 0.0004, and 72 hpi; *q* < 0.0054). There have been studies that ions such as calcium and iron level in host intracellular can enhance the infective capacity of the *T. cruzi*. It suggests that AS in genes involved in iron metabolism can be modulated in host cells during T. cruzi infection cycle^42,43^.

Furthermore, we showed that how *HDAC7* exon skipping can act as a regulator to dynamically control host cell cycle pathway during intracellular infection. Especially, the original study reported that the ten cycle genes were down-regulated at 4 and 24 hpi but up-regulated at 72 hpi at a gene expression level^19^. These dynamic changes may be controlled by the exon skipping of *HDAC7*. In contrast to the previous gene expression analysis showing up-regulation of the cell cycle genes in early infection phase^19^, we did not find the significantly overrepresented cell cycle pathway in the early-mid phase (i.e. 4 and 24 hpi), and it suggests that cell cycle genes in early phase may be mainly affected by gene expression change rather than AS alteration. Methylation, an epigenetic factor, is one of the host cell environmental factors affected by infection. It is known to be connected with splicing regulation in that MECP2 binding to hypo-methylated sequences may cause a temporal pause of the transcription process that helps promote correct splicing^44^. The role of methylation in regulating splicing reflects the differential distribution of GC content across exons and introns; for example, constitutive exons tend to have higher GC content than do AS exons, leading to a lower chance for AS exons to be methylated^45^. Thus, methylation status is another factor that may affect splicing frequencies.

We further analyzed data from an independent cohort as partial replication of our results. This independent data was generated from a study investigating genes differentially expressed in human HFF cells infected with virulent and non-virulent strains of *T. cruzi*^46^. Of this dataset, we investigated RNA-seq data obtained from control (uninfected) HFF cells (n = 4) and those infected by CL Brener (n = 3), which is a virulent reference clone from the *T. cruzi* genome project, collected 60 h post infection (hpi). Of available datasets, this one was the most relevant to our first dataset in terms of the host cell type and timepoint after infection; none matched the exact experimental design used in the first dataset. We processed the RNA-seq data using the same criteria as in the previous analysis (see “Methods” section). As shown in Supplementary Fig. S4, we observed similar distributions of PSI levels for the 15th exon of *HDAC7* in uninfected control samples for both our previous analysis (Y strain, solid lines) and the independent cohort analysis (CL Brener, dashed lines). In addition, the PSI level of the *HDAC7* 15th exon at 60 hpi in CL Brener-infected HFF cells was similar to the pattern observed in our previous analysis over time (i.e. PSI similar to the value at 72 hpi). In addition, we compared the PSI levels of AS exons previously found to be differentially expressed in the later infection phase at 72 hpi (Y strain) to those of AS exons at 60 hpi (CL Brener), and obtained a correlation efficiency of 0.7 (Supplementary Fig. S5).

However, it is worth mentioning limitations in this study. We analyzed the very small sample size for each timepoints (n = 3–4), which may have contributed to the lack of strongly significant enriched functions in early infection. Even though we verified our study with partial replication in an independent dataset, there may still be a limitation that we did not validate our result with either an experiment or independent cohort data which is exactly matched to the data from our main analysis in this study. However, we found evidence that AS exons may be regulated during the infection, and these genes are worthy of further experimental validation or replication with independent data sets.

## Conclusion

In conclusion, our bioinformatics analysis of transcriptome found evidence of transcriptomic regulation via AS resulting from the interaction between *T. cruzi* and its host cell, and these are worthy to be experimentally validated to better understand the interaction. Thus, these results can be a useful basic resource for further study in potentially assisting identify molecular candidates for improving treatment of *T. cruzi* infection.

## Methods

The overall methods for identifying differentially expressed AS exons are depicted in Supplementary Fig. S6. RNA-Seq data was obtained for four timepoints, 4 (n = 3), 28 (n = 3), 48 (n = 4), and 72 (n = 3) hpi, along with one uninfected control group (n = 7). We used rMATs to estimate the PSI value of each exon based on the ratio of IJ (inclusion junction) reads to the total reads [i.e. sum of IJ and SJ (skipped junction)], as described in Supplementary Fig. S6. Differential AS events were defined as significant with a cutoff at FDR < 0.05 and PSI > 10%. Our methods are as follows.

### RNA-Seq data analysis

We downloaded previously published RNA-Seq data (Project ID SRP043008) from the NCBI (https://www.ncbi.nlm.nih.gov/) Sequence Reads Archive (SRA). This project comprises 101 nt paired-end reads generated from HFFs infected by *T. cruzi* (Y strain) at multiple time points (4 hpi; n = 3, 24 hpi; n = 3, 48 hpi; n = 4, and 72 hpi; n = 3) and control uninfected cells (n = 7). The original publications generated RNA-seq data from HFF BJ cell line (ATCC CRL-2522). From each time point, biological replicates were generated independently^29^. We first converted the SRA format to raw FASTQ using fastq-dump.2 from the SRA Toolkit (v.2.8.0, https://ncbi.github.io/sra-tools/). We evaluated the quality of whole reads using the FastQC package v0.11.4^47^ for a quality control. We then mapped the reads to the human reference genome (GRCh 37.75 based on hg19) using the STAR (v2.5) aligner^48^ with splicing annotation information from the reference in GTF format. We selected reads that were uniquely mapped and properly paired using samtools (v. 1.5)^49^.

### Identification of differentially expressed AS exons

Our analysis considered four AS exon events: exon skipping (ES), alternative 3′ splice site (A3SS), alternative 5′ splice site (A5SS), and intron retention (IR)^50^. We used rMATs (v3.2.5)^51^ to identify each AS event based on the GTF and estimate the PSI for each sample. PSI indicates the fraction of mRNA that include the AS exon and can be estimated as the ratio of reads with included and skipped exons^51–53^. Next, we compared PSI levels between control cells and each post-infection timepoint (i.e. control vs. 4hpi, control vs. 12 hpi, control vs. 48 hpi, and control vs. 72 hpi). AS exons were considered significant at cutoff values of FDR < 0.05 and PSI > 0.1 (10% difference between groups). In order to verify whether the PSI levels were informative for stratifying cells within each time course, we carried out a principle component analysis on the PSI levels of all samples using the PCA function in R (v. 3.4.4).

### Functional enrichment analysis

For genes identified as having differential AS exons (defined as AS genes), we interpreted their functional roles in the parasite life cycle by performing enrichment analysis with ConsensusPathDB (Release 33, CPDB; https://cpdb.molgen.mpg.de/)^54^. Enrichment of pathways and Gene Ontology (GO) terms was considered significant at an adjusted *q* value < 0.05. We reduced redundancy and removed potential false positive GO terms using the GO-module web-based tool (https://lussierlab.org/GO-Module) (v.1.3)^55^. To reduce a redundancy of GO terms with similar functions, we selected most significant GO terms.

### Hierarchical clustering of the identified differential AS exons

To reveal any longitudinal patterns in AS, we performed a hierarchical clustering analysis on the PSI values of differential AS exons over time using pheatmap (R package, https://cran.r-project.org/web/packages/pheatmap/index.html), and visualized the results as a heatmap. In addition, we performed k-means clustering, which separated the genes into seven sub-clusters with similar expression profiles across different developmental timelines of AS exons. We used R function “kmeans” of “STAT” R package (https://cran.r-project.org/web/packages/STAT/index.html) with the Hartigan-Wong algorithm. Then, we performed functional GO analysis with ConsensusPathDB (Release 33, CPDB; https://cpdb.molgen.mpg.de/)^54^.

### Identification of trans-acting regulators (splicing factors) affecting AS exons

Given the knowledge that splicing factors control AS efficiency, we performed a linear regression between the gene expression of known splicing factors and PSI levels of exons with significant splicing events at any timepoint. We obtained a list of 280 splicing factor genes from the Bioconductor package TIN^56^, and their gene expression values, as normalized count per million values (CPM), were obtained from “S3 Table” in the original study^29^.

### Case study: HDAC7

We performed functional annotation of splicing event of *HDAC7* on *T. cruzi* life cycle during intracellular infection as a case study. We visualized how *HDAC7* exon 15 potentially affects protein function by predicting 3D structures of translated amino acid sequences from the canonical using I-TASSER (https://zhanglab.ccmb.med.umich.edu/I-TASSER/), and visualized them using PyMol (v1.3, https://pymol.org). To evaluate possibility of *HDAC7* as a regulator, we identified genes whose expression levels were associated with the *HDAC7* 15th exon skipping, and then evaluated their functional roles. Associated genes were identified by performing a linear regression and Pearson correlation test between each expression of all protein coding genes (20,758) and the PSI value of the 15^th^ exon of *HDAC7*. We used the gene expression levels that were estimated as normalized CPM (count per million values) by the original study^19^. Associations were considered significant if they had an adjusted FDR < 0.05 and an absolute correlation value > 0.9. We then defined functional roles of significantly associated genes through enrichment analysis with CPDB. For significant genes, we visualized their expression level as a heatmap using the R package *pheatmap* (v.1.0.8, https://cran.r-project.org/web/packages/pheatmap/index.html).

## Supplementary information

Supplementary Figures.

Supplementary Table S1.

Supplementary Table S2.

Supplementary Table S3.

Supplementary Table S4.

Supplementary Table S5.

## Data Availability

All data used in this study were obtained from public datasets (https://www.ncbi.nlm.nih.gov/sra/SRP043008)^20,29^.
