# Supplementary material for: Transcriptome analysis of alternative splicing in the pathogen life cycle in human foreskin fibroblasts infected with Trypanosoma cruzi
Source: Sci Rep. 2020 Oct 15;10:17481. doi: 10.1038/s41598-020-74540-9 (PMC7566602; doi:10.1038/s41598-020-74540-9)
Supplement: Supplementary file 1 — Supplementary Figures. [file 41598_2020_74540_MOESM1_ESM.pdf]

Supplementary data 1

Transcriptome analysis of alternative splicing in the pathogen life cycle in human foreskin fibroblasts infected with *Trypanosoma cruzi*

Hyeim Jung [1, †], Seonggyun Han [2, †], and Younghee Lee[2,*]

[1] Department of Internal Medicine, Washington University School of Medicine, St. Louis, MO 63110, USA. [2]Department of Biomedical Informatics, University of Utah School of Medicine, Salt Lake City, UT, USA.

[†] Contributed equally

Corresponding Authors: Younghee Lee, Ph.D.

Corresponding authors' address: Younghee Lee
Department of Biomedical Informatics
University of Utah School of Medicine
Salt Lake City, Utah, USA
younghee.lee@utah.edu

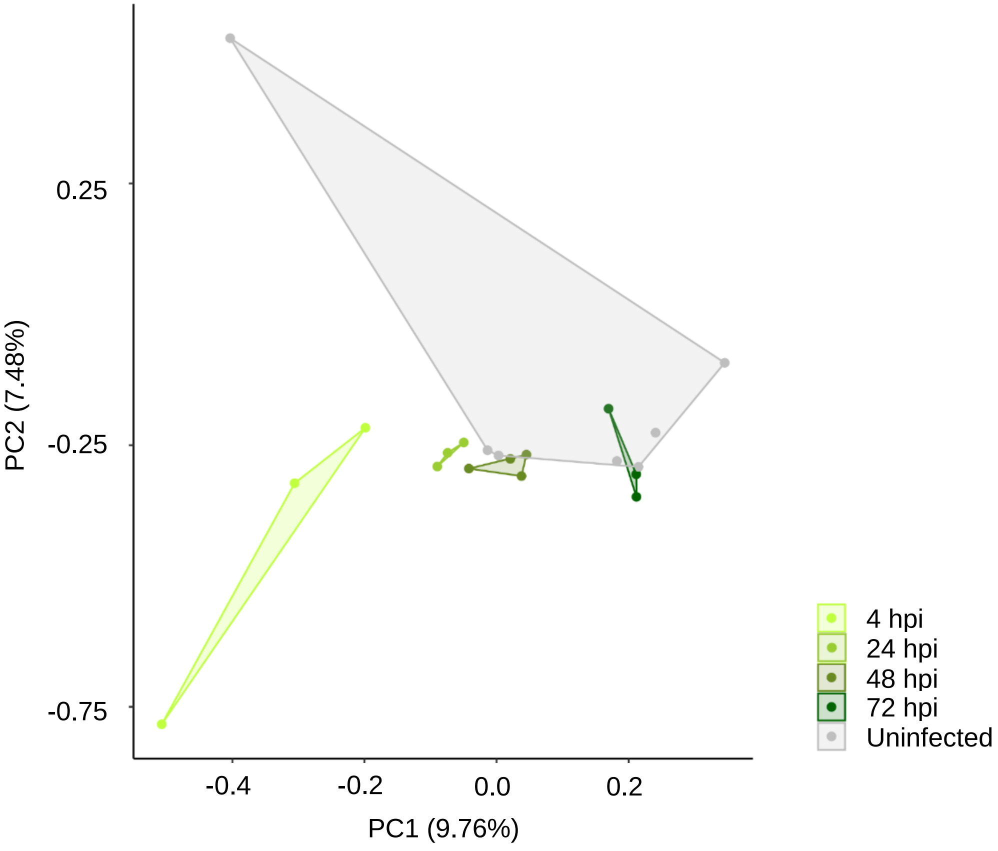

Supplementary Fig. S1. Principal component analysis of all AS exons. Each dot indicates an individual sample. Grey indicates uninfected samples and green infected samples; color darkness indicates the infection phase.

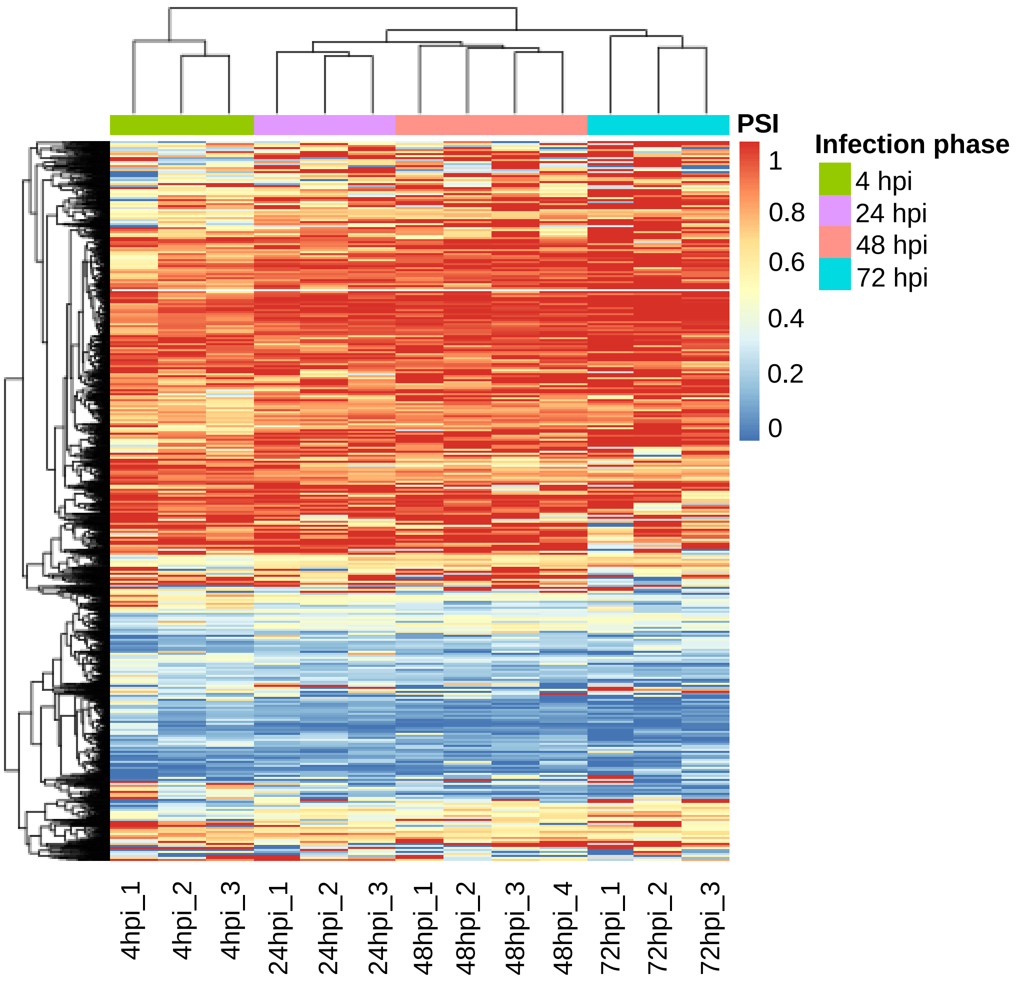

Supplementary Fig. S2. A heatmap including hierarchy based on PSI levels. Each row refers to a given exon and each column indicates a sample. The samples are clustered according to post-infection timepoint.

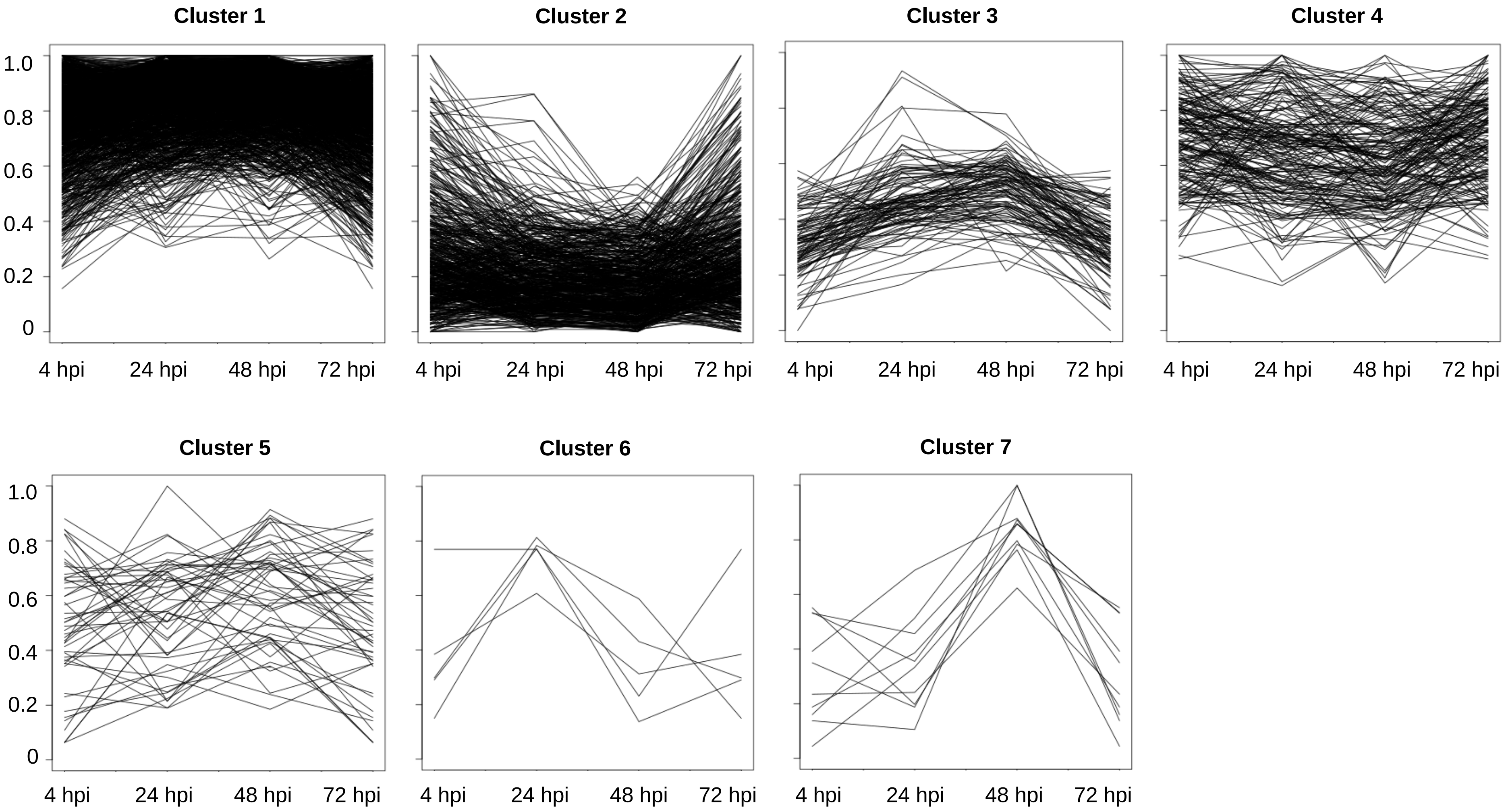

Supplementary Fig. S3. Longitudinal patterns in PSI values over time. Each plot presents a sub-cluster generated by k-means clustering. X-axis refers to the timepoint, and the y-axis indicates PSI levels.

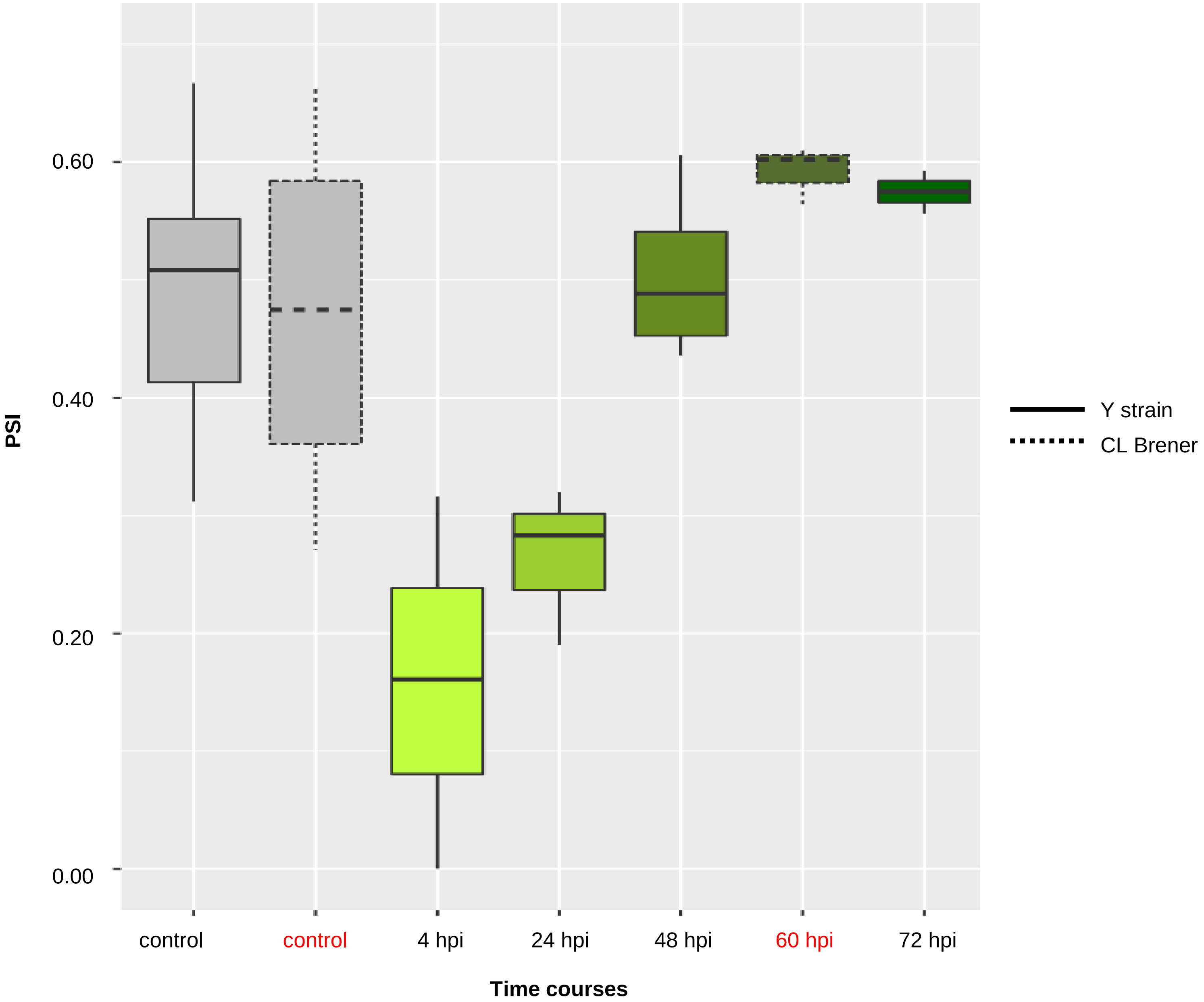

Supplementary Fig. S4. Boxplot of PSI levels of exon 15 according to treatment group (control and various time courses). PSI level indicates inclusion level of the 15th exon. The shade of color corresponds to timepoint. Solid and dashed lines refer to the Y strain (previous data) and CL Brener strain (independent cohort data) of T. cruzi, respectively.

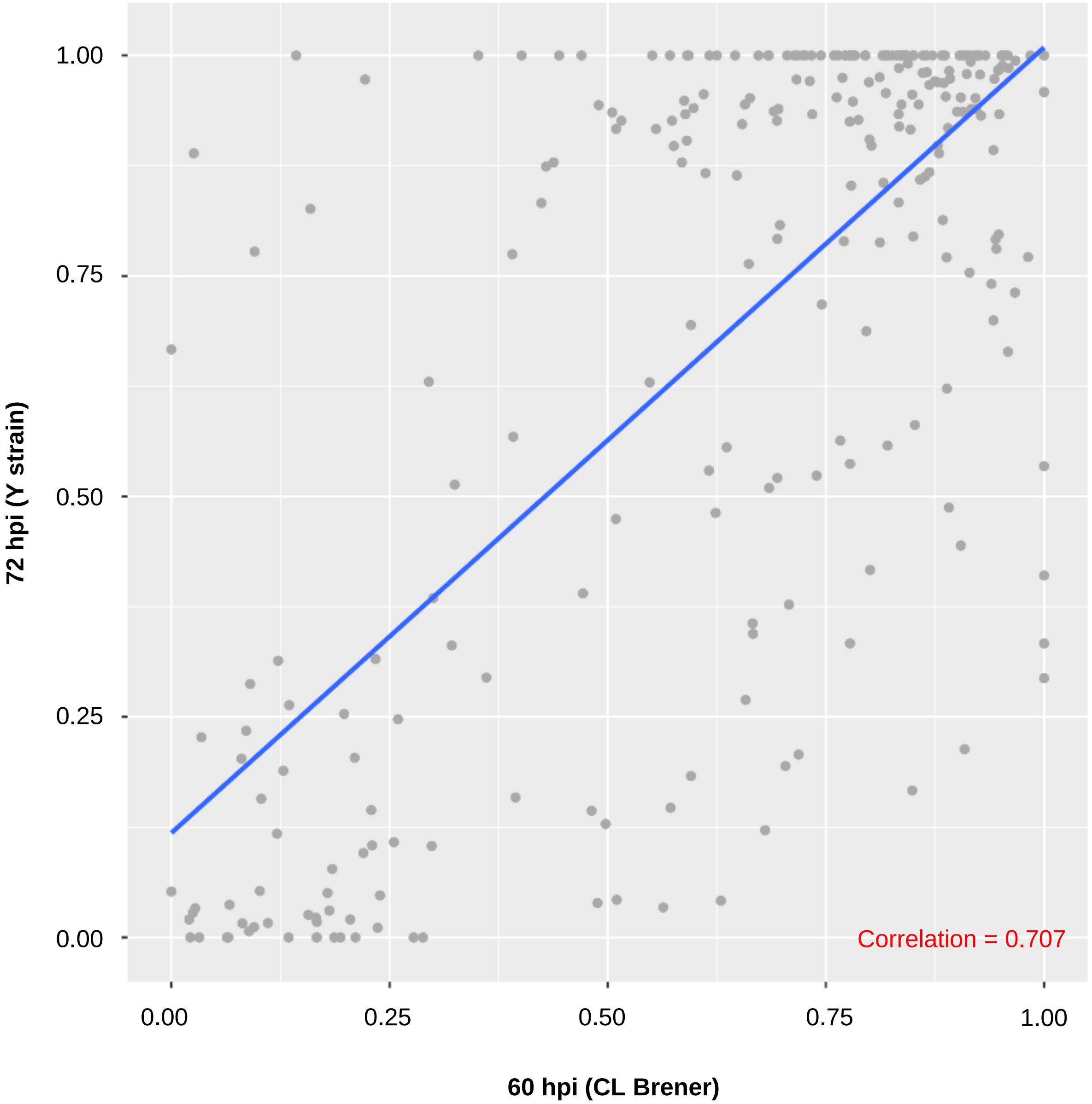

Supplementary Fig. S5. Scatterplot of PSI levels of differential AS exons discovered at 72 hpi in Y strain-infected HFFs. X-axis and Y-axis indicate PSI status at 60 hpi with CL Brener and 72 hpi with Y strain, respectively.

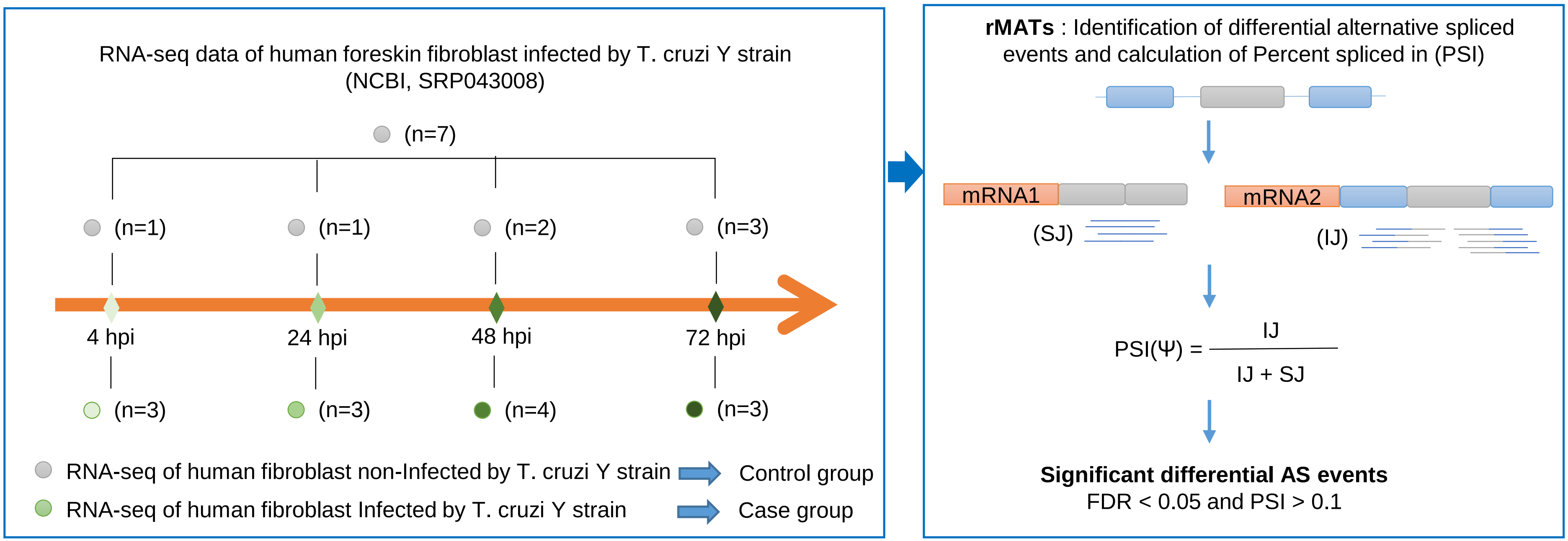

Supplementary Fig. S6. The overall process for identifying alternatively spliced exons in infected HFF cells. We mapped RNA-Seq reads (SRP id = SRP043008) to the human reference genome (hg19). We then estimated the PSI values of AS exons in each time point using rMATs and compared identified differentially expressed AS exons. Significance cut-off values were FDR < 0.05 and PSI > 0.1, indicating 10% difference.
